# CVD Synthesis of Intermediate State-Free, Large-Area and Continuous MoS_2_ via Single-Step Vapor-Phase Sulfurization of MoO_2_ Precursor

**DOI:** 10.3390/nano11102642

**Published:** 2021-10-08

**Authors:** Tinna Chiawchan, Harihara Ramamoorthy, Kanokwan Buapan, Ratchanok Somphonsane

**Affiliations:** 1 Department of Physics, Faculty of Science, King Mongkut’s Institute of Technology Ladkrabang, Bangkok 10520, Thailand; 62605032@kmitl.ac.th (T.C.); 62605033@kmitl.ac.th (K.B.); ratchanok.so@kmitl.ac.th (R.S.); 2 Department of Electronics Engineering, Faculty of Engineering, King Mongkut’s Institute of Technology Ladkrabang, Bangkok 10520, Thailand; 3 Thailand Center of Excellence in Physics, Commission on Higher Education, 328 Si Ayutthaya Road, Bangkok 10400, Thailand

**Keywords:** 2D materials, monolayer MoS_2_, chemical vapor deposition, Raman, MoO_2_ precursors

## Abstract

The low evaporation temperature and carcinogen classification of commonly used molybdenum trioxide (MoO_3_) precursor render it unsuitable for the safe and practical synthesis of molybdenum disulfide (MoS_2_). Furthermore, as evidenced by several experimental findings, the associated reaction constitutes a multistep process prone to the formation of uncontrolled amounts of intermediate MoS_2−y_O_y_ phase mixed with the MoS_2_ crystals. Here, molybdenum dioxide (MoO_2_), a chemically more stable and safer oxide than MoO_3_, was utilized to successfully grow cm-scale continuous films of monolayer MoS_2_. A high-resolution optical image stitching approach and Raman line mapping were used to confirm the composition and homogeneity of the material grown across the substrate. A detailed examination of the surface morphology of the continuous film revealed that, as the gas flow rate increased by an order of magnitude, the grain-boundary separation dramatically reduced, implying a transition from a kinetically to thermodynamically controlled growth. Importantly, the single-step vapor-phase sulfurization (VPS) reaction of MoO_2_ was shown to suppress intermediate state formations for a wide range of experimental parameters investigated and is completely absent, provided that the global S:Mo loading ratio is set higher than the stoichiometric ratio of 3:1 required by the VPS reaction.

## 1. Introduction

In the past few years, 2D-MoS_2_ has emerged as one of the most popular candidates among the 2D-TMDC family of materials. In contrast to graphene, 2D-MoS_2_ is a semiconductor with a non-zero bandgap, which allows for the realization of transistors with high on–off current ratios [1]. Moreover, the bandgap is tunable via modulation of the layer thickness, changing from an indirect-to-direct bandgap as the thickness approaches the monolayer limit [2,3]. The latter feature is especially beneficial for realizing optoelectronic applications, such as photodetectors and photo-emitters. As expected, the increased popularity of 2D-MoS_2_ has led to several demonstrations of FETs, photodetectors and sensors, as evident from the literature [4,5,6,7,8,9,10,11,12,13,14,15].

Furthermore, 2D-MoS_2_ can be synthesized by using either top-down approaches, such as chemical or mechanical exfoliation, or bottom-up synthesis routes, such as chemical vapor deposition (CVD). While mechanical exfoliation has been extensively used due to the resulting high sample quality, the obtained crystal sizes are random in nature, and the overall yield is often quite limited. This makes it difficult to extend their usability to practical applications. In comparison to exfoliation, CVD growth methods have showed great promise in terms of improved yield, scalability and homogeneity of the grown films, making it the method of choice for implementing future technology that could be made viable through integration with the current CMOS platform. However, to truly unlock the potential of 2D-MoS_2_ as a replacement material for conventional silicon, it is necessary to be able to produce large-scale, defect-free and continuous MoS_2_, using a low-cost and safe approach.

CVD-grown MoS_2_ has been synthesized via the sulfurization of Mo containing precursors, such as Mo [16], MoO_2_ [17,18,19,20] and MoO_3_ [21,22,23,24,25,26,27,28,29,30,31,32,33,34,35,36,37,38,39,40,41,42,43,44,45,46,47,48,49], among which the use of MoO_3_ powder as the precursor has remained the most popular method owing to the possibility of obtaining large area single crystal and also continuous films [41,42,43,44,45,46,47,48,49].In fact, in very recent work, Q. Wang et al. [46] have employed a customized multisource CVD system to successfully demonstrate the wafer-scale growth of MoS_2_ using MoO_3_ as the precursor material. While these accomplishments are noteworthy, the poisonous nature (strong irritant and carcinogen according to GHS classification) and low evaporation temperature (350 °C) of MoO_3_ powder means that harmful exposure to this material is more likely, posing a serious health hazard situation. A safer material choice is therefore needed for both small-scale research purposes and meeting large-scale production needs.

In a previous report, Bilgin et al. [17] raised an important question of whether the use of MoO_3_ as a precursor material is really necessary when MoO_2_, a more stable oxide, could be used instead. In their work, a direct vapor-phase sulfurization (VPS) of MoO_2_, which is characterized by a single-step chemical reaction ${\mathrm{MoO}}_{2}\;+\;3S\to {\mathrm{MoS}}_{2}\;+\;{\mathrm{SO}}_{2}$, was employed to successfully grow MoS_2_ crystals. The crystals were shown to be not only possible to grow on a wide range of substrates such as Si, Si_3_N_4_, SiO_2_, quartz and mica, but were also of high optoelectronic grade, as assessed from the excitonic states observed from photocurrent spectroscopy measurements [17].

It is widely accepted that when MoO_3_ is used as the initial precursor, the final MoS_2_ product results from a two-step process: the first being the reduction of MoO_3_ to MoO_2_ (Equation (1)) which is then followed by a second step which involves the sulfurization of MoO_2_ (Equation (2)) [17,41].

(1) $${\mathrm{MoO}}_{3}\;+\;\frac{x}{2}S\to {\mathrm{MoO}}_{3-x}\;+\;\frac{x}{2}{\mathrm{SO}}_{2}$$

(2) $${\mathrm{MoO}}_{3-x}\;+\;\frac{7-x}{2}S\to {\mathrm{MoS}}_{2}\;+\;\frac{3-x}{2}{\mathrm{SO}}_{2}$$

As is evident from these equations, an ideal situation (x = 1) would result in the intermediate compound being MoO_2_. However, since growth conditions within a CVD furnace are rarely ideal, it was suggested that incomplete reactions could result in varying amounts of MoS_2−y_O_y_ phase [17,50,51]. This “phase-mixing” with the MoS_2_ has been frequently observed in experiments [23,25,48,52] and remains an important issue that needs to be addressed.

Recently, through detailed experimental work varying the S:Mo molar ratio, the stepwise sulfurization of MoO_3_ to MoS_2_ via an intermediate reaction forming molybdenum oxysulfide (MoOS_2_) was reported [36]. The authors of this work have proposed the following reaction for the formation of MoOS_2_ from the intermediate phase MoO_2_.

(3) $$2{\mathrm{MoO}}_{2}\left(v\right)\;+\;5S\left(v\right)\to 2{\mathrm{MoOS}}_{2}\left(s\right)\;+\;{\mathrm{SO}}_{2}\left(v\right)$$

The above reaction pathway suggests that, even in an ideal scenario (x = 1), where MoO_3_ is appropriately reduced to MoO_2_, the formation of intermediate states may not be fully avoidable.

The role of MoO_2_ as an intermediate phase was further investigated in a different study conducted by Hyun et al. [35]. Here, after the synthesis of MoS_2_, it was found that the MoO_3_ powder used for the growth was reduced by sulfur to form MoO_2_ powder. In addition, MoO_2_ crystals were also reported to be formed on the growth substrate. These findings were explained by proposing a thermodynamically favorable (negative Gibbs free energy change) reaction pathway (Equation 4) for the formation of solid-phase MoO_2_ from MoO_3_ powders [35].

(4) $$4{\mathrm{MoO}}_{3}\left(s\right)\;+\;S_{2}\left(v\right)\to 4{\mathrm{MoO}}_{2}\left(s\right)\;+\;2{\mathrm{SO}}_{2}\left(v\right)$$

Based on these observations, it was proposed that when using MoO_3_ powders as the precursor, a much more complex scenario could arise wherein the final MoS_2_ could potentially be synthesized by a number of thermodynamically favorable pathways. These include the sulfurization of vapor-phase MoO_3_, the sulfurization of intermediate solid-phase MoO_2_ state and finally the sulfurization of vapor-phase MoO_2_, resulting from the vaporization of the reduced MoO_2_ powder (see Equations (5)–(7)) [35].

(5) $$4{\mathrm{MoO}}_{3}\left(v\right)\;+\;7S_{2}\left(v\right)\to 4{\mathrm{MoS}}_{2}\left(s\right)\;+\;6{\mathrm{SO}}_{2}\left(v\right)$$

(6) $$4{\mathrm{MoO}}_{2}\left(s\right)\;+\;6S_{2}\left(v\right)\to 4{\mathrm{MoS}}_{2}\left(s\right)\;+\;4{\mathrm{SO}}_{2}\left(v\right)$$

(7) $$4{\mathrm{MoO}}_{2}\left(v\right)\;+\;6S_{2}\left(v\right)\to 4{\mathrm{MoS}}_{2}\left(s\right)\;+\;4{\mathrm{SO}}_{2}\left(v\right)$$

Importantly, while all three proposed reactions are thermodynamically favorable with a negative Gibbs free energy change value, the reaction (Equation (7)) involving the direct VPS of MoO_2_ was found to have the most negative value for the Gibbs free energy change value, making it the most thermodynamically favorable reaction. This is followed by the VPS of MoO_3_ (Equation (5)), and finally the solid-phase sulfurization of MoO_2_ (Equation (6)) taking the highest Gibbs free energy change values [35].

It is clear from the thermodynamic considerations discussed thus far that a single-step VPS reaction of MoO_2_ should provide the best chemical pathway to synthesize MoS_2_ after taking into account the associated reaction simplicity, safety and ability to greatly suppress intermediate state formations if not fully eliminate it. Despite this, there have been only a few experimental studies dedicated towards evaluating the potential of directly using MoO_2_ as a precursor. Following the experimental works of Bilgin et al. [17], Xie et al. [18] studied the effect of growth parameters such as the S:Mo molar ratio and the introduction time of the sulfur for initiating MoS_2_ growth. These parameters were shown to affect the MoS_2_ domain shape which was found to be tunable from triangles to hexagons. While large area growth (centimeter scale) was reported, the MoS_2_ obtained were discreet single crystal domains and not continuous films. It was also suggested that the lower evaporation ability of MoO_2_ inhibits the coalescence of flakes and therefore also the probability of forming large-area continuous films.

In this work, we successfully grow centimeter-scale continuous films of monolayer MoS_2_ using the single-step VPS method prescribed by Bilgin et al. [17] We find that the final growth pattern takes the form of distinct parabolic growth zones containing varying morphology of the synthesized MoS_2_, which is typical of the presence of a concentration-gradient of the Mo-source across the growth substrate. Different from prior works, we employed a high-resolution optical image stitching approach to create a clear map of the growth zones, as well as to capture the area coverage of the continuous monolayer MoS_2_ typically found in the downstream zone. Detailed Raman point analyses, along with area and line scan mappings, are performed to confirm the material composition of the various zones and the homogeneity of the continuous films. The effect of varying gas flow rate in our experiments is carefully studied, and we find striking dependence of the morphology and quality of the grown films on this parameter, as determined using scanning electron microscopy (SEM). In particular, the increase of gas flow rate drives the CVD reaction from a thermodynamically stable regime to a kinetically unstable one and results in the drastic reduction in grain boundary separations of the continuous films. Additionally, by closely studying the effects of other growth parameters such as the MoO_2_ weight, S:Mo molar ratio and crystal growth time, we show that there are striking similarities in the growth results when compared to the results obtained from the conventional growth process employing MoO_3_ precursors.

The most significant finding of our work is that the synthesized MoS_2_ is found to be completely free of intermediate reaction by-products (such as MoO_2_ and MoOS_2_), as long as the S:Mo loading ratio is set above the stoichiometric value of 3:1 fundamentally required by the direct VPS reaction of MoO_2_ to properly form MoS_2_. For S:Mo ratios greater than 3:1, even the thickest material found on our growth substrate corresponds to fully sulfurized bulk MoS_2_ and not of any intermediate states, which strongly proves the overall effectiveness of using the single-step direct VPS of MoO_2_. This is in striking contrast to past reports employing MoO_3_ as the precursor, where intermediate states were a common observation, even as the S:Mo ratios were set at values orders of magnitude in excess of the stoichiometric requirements. We therefore believe that the results presented here should strengthen previous assertions of the logical choice of MoO_2_ as the precursor material and provide a platform for further exploration of the wafer-scale growth of continuous and homogenous MoS_2_ films, a prospective that holds tremendous promise for the future of semiconductor technology and CMOS scaling.

## 2. Materials and Methods

### 2.1. CVD Synthesis of MoS_2_ Using Direct MoO_2_ Precursor

**Figure 1:**
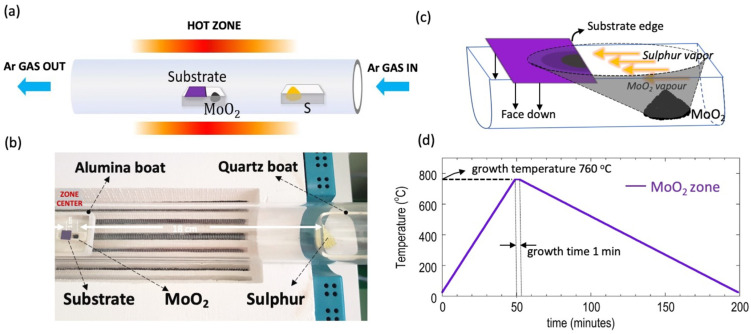
(**a**) Schematic of the CVD setup used for the growth of MoS_2_ using MoO_2_ as the precursor. (**b**) Example photograph of the heating zone taken prior to starting the growth. (**c**) Schematic showing the zonal growth pattern of MoS_2_ initiating from the substrate edge. (**d**) Temperature profile of the heating zone where the MoS_2_ growth process takes place. Photograph courtesy of Harihara Ramamoorthy, Copyright 2021.

Centimeter-scale continuous films of MoS_2_ reported in this work were synthesized using a simple atmospheric pressure CVD scheme similar to Bilgin et al. [17], with few changes. The CVD system consists of a single heating zone and a quartz tube of 50 mm diameter inside which the MoS_2_ growth takes place at a growth temperature of 760 °C. A 300 nm thermal oxide Si/SiO_2_ wafer is firstly cut into ~10 mm × 10 mm squares and subsequently cleaned with 1 min of ultrasonication in acetone, followed by an IPA rinse and finally blown dry with nitrogen gas. Without any further surface processing, the cleaned wafer is centered face-down on a small (26 mm × 8 mm × 7 mm) alumina boat preloaded with the required amount of MoO_2_ precursor (99% purity Alfa Aeser, Haverhill, MA, USA) placed at the upstream end of the boat, approximately 1 cm from the center of the growth substrate (see Figure 1a,b). The sample-and-precursor-containing boat is then placed on a second alumina boat, and the assembly is carefully inserted into the quartz tube, such that the substrate position aligns with the center of the heating zone. In a different quartz boat, the required amount of sulfur powder (99.5% Alfa Aeser, Haverhill, MA, USA) is loaded such that the sulfur is at the edge (approximately 18 cm from the center) of the heating zone (see Figure 1b). The tube is then purged with a 2000 sccm of Ar gas (>99.9% purity) for 5 min at room temperature. Following this, the flow rate is lowered to a value required by the experiment and the temperature of the heating zone is ramped up at the rate of 15 °C/min until the required growth temperature is reached and subsequently held here for the required growth time as designed by the experiment. At the end of the growth cycle, the power to the furnace is turned off and the sample is allowed to naturally cool down to room temperature. Figure 1d shows the typical growth zone temperature profile used in this work.

### 2.2. MoS_2_ Characterization

The quality and thicknesses of the grown MoS_2_ material was assessed by using optical imaging, micro-Raman spectroscopy, AFM and SEM analysis. Optical imaging and high-resolution image stitching were obtained by using a CX-40M microscope from Ningbo Sunny Instruments, Co., Ltd., Yuyao, China. The micro-Raman spectra were obtained from a Horiba LabRAM HR Evolution confocal Raman system (Horiba Ltd., Bangkok, Thailand) at a laser excitation wavelength of 532 nm. AFM was performed by using a AFM5500M system from Hitachi High-Tech Ltd., Bangkok, Thailand. A SU8030 Field-Emission Scanning Electron Microscope (FESEM) from Hitachi High-Tech Ltd., Bangkok, Thailand was used to analyze the surface morphology of the grown MoS_2_ films.

## 3. Results and Discussions

The quantity of active Mo species accessible at the substrate surface for nucleation and subsequent domain formation play a crucial role in the entire growth process of MoS_2_. While a uniform distribution of gaseous phase S is typically achieved by maintaining a large distance of separation between the S source and the growth substrate (~18 cm in our case), the Mo-containing source is typically placed in close proximity to the growth substrate. This results in a prominent gradient-like concentration distribution of the MoS_2_ nucleation sites at the substrate surface, which then serves as the blueprint for the final growth pattern (see schematic shown in Figure 1c). Numerical simulations performed by R. A. Vila et al. [53] using finite element modelling revealed a decreasing concentration gradient on the surface of the substrate as the distance from the Mo-source increases, and that the concentration gradient pattern is characterized as distinct parabolic growth zones commonly found in experiments [24,29,36,45,49]. Moreover, the presence of such a concentration gradient means that the local Mo:S ratio varies along the length of the growth substrate, increasing the probability of morphology evolutions [53], oxysulfide formations [33,36,48,50,51,52] and MoS_2_ domain-shape changes [24]. To make matters worse, experimental factors, such as precursor amount, gas flow rate, substrate position relative to the MoO_3_ source, growth temperature, geometrical parameters and possibly the spatial distribution of the MoO_3_ precursor itself, all have an impact on the Mo flux arriving at the substrate.

### 3.1. Zonal Deposition Pattern of MoS_2_

**Figure 2:**
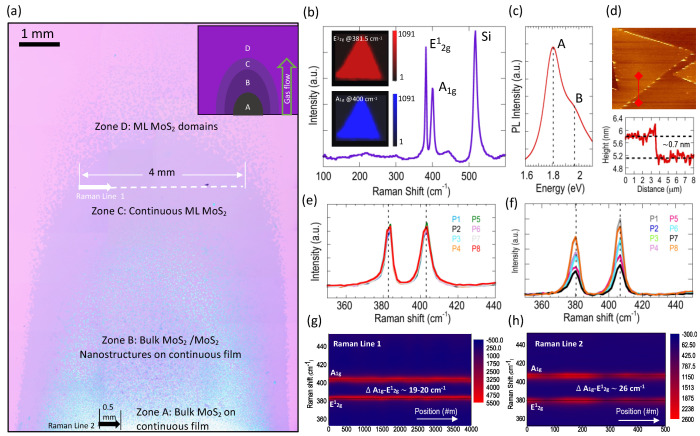
(**a**) High-resolution optical stitched image revealing the gradient pattern of MoS_2_ growth obtained for the following growth parameters: MoO_2_ = 3 mg, S = 300 mg, flow rate = 10 sccm, growth temperature = 760 °C and growth time = 1 min. The various zones A–D are indicated, along with the type of material found to be grown in the respective zones. Location where the Raman line maps shown in panels (**g**,**h**) were acquired, are also indicated. (**b**) Raman spectra acquired over a wide spectral range for a MoS_2_ domain found in zone D. The inset shows Raman maps obtained for the same MoS_2_ domain at the E^1^_2g_ (top) and A_1g_ (bottom) frequency. (**c**) Photoluminescence (PL) trace obtained for monolayer MoS_2_ domain showing the characteristic A and B excitonic peaks. (**d**) AFM image (top) of a MoS_2_ domain found in zone D and the height profile (bottom) obtained along the red line marked in the AFM image. (**e**,**f**) Raman spectra collected at eight locations from the line map shown in panels (**g**,**h**) respectively. (**g**) Raman line scan mapping obtained along the white dashed line indicated in panel (**a**) for the monolayer and continuous MoS_2_ grown in zone C. (**h**) Raman line scan mapping obtained along the black line indicated in panel (**a**) for the thick MoS_2_ grown in zone A.

The aforementioned scenarios should also be expected when using MoO_2_, which is the precursor employed in our work. As a result, the formation of distinct growth zones can be expected, as shown in the schematic of Figure 1c, with each zone containing growth crystal with potentially varied characteristics. Experimentally, this can be directly seen in Figure 2a, where we show the MoS_2_ growth results obtained by using the following set of growth parameters: MoO_2_ amount = 3 mg, S amount = 300 mg, gas flow rate = 10 sccm, growth temperature = 760 °C and growth time = 1 min. The high S:Mo loading ratio of 100:1 is initially chosen to ensure complete sulfurization of MoO_2_ whereas the short growth time prevents the formation of nanoparticles on the MoS_2_ domains and thin films. The effects of these growth factors are investigated in detail, and they are discussed in the later sections.

Unlike previous reports, the high-resolution optical image stitching approach used in our work aids in the visualization of MoS_2_ film growth and its coverage across the entire growth substrate. As seen in Figure 2a, the image stitching reveals very clearly the formation of the four distinct, parabolic-shaped growth zones, A to D, marked by the dotted lines. Zone A which is closest to the substrate edge is dominated by a thick white-colored material. Zone B is seen to contain a mix of the white material (seen in Zone A) and “rod-like” nanostructures (discussed later). Zone C, as judged from the uniform optical contrast, represents a continuous layer of monolayer MoS_2_ and finally zone D is dominated by the formation of individual monolayer MoS_2_ domains.

Figure 2b shows the Raman spectra, obtained over a wide spectral range, for a MoS_2_ domain selected from a random location in zone D and in close proximity (~100 μm) to the boundary separating zones C and D. The two dominant Raman vibration modes which are characteristic of MoS_2_ can be seen here. The E^1^_2g_ mode, corresponding to the in-plane vibration of S and Mo atoms, is seen at a wavenumber of approximately 381.5 cm^−1^ and the A_1g_ mode, corresponding to the out-of-plane vibrations of S atoms, is seen at about 400 cm^−1^. The resulting frequency difference (Δω) between the two modes is therefore ~18.5 cm^−1^, confirming the monolayer nature of the MoS_2_ domain [54,55]. In addition to the two Raman modes of MoS_2_, a third vibration peak corresponding to that of Si is observed at a wavenumber of 516 cm^−1^. The thickness uniformity of the MoS_2_ domain is further analyzed by using Raman mapping, the results of which are shown in the inset of Figure 2b. It is evident from the uniform Raman intensity maps, obtained at both vibration modes (E^1^_2g_ and A_1g_), that the thickness of the MoS_2_ domains grown in zone D is highly uniform. Figure 2c displays the photoluminescence (PL) spectra obtained for a monolayer MoS_2_ domain found in zone D. The characteristic A and B direct excitonic transitions that occur at ~1.81 and ~1.95 eV, respectively, and the relatively large A peak intensity, attest to the monolayer nature and the good crystalline quality of the grown MoS_2_ [2,17].

We additionally performed AFM imaging (Figure 2d), and the corresponding step height measurements revealed a thickness of ~0.7 nm. This value is in close agreement with the structural thickness of 0.615 nm for an isolated S-Mo-S slab, and therefore confirms the monolayer characteristics of the grown MoS_2_ triangles. To study the characteristics of the continuous MoS_2_ film grown in zone C, we performed an elaborate 25-point Raman line scan mapping (see Figure 2g) over a distance of 4 mm, marked by the white dotted line shown in Figure 2a. The film homogeneity is immediately apparent from the relatively unchanging E^1^_2g_ and A_1g_ positions across the mapped line. The line scans revealed a frequency difference lying in the 19–20 cm^−1^ range, which confirms the monolayer nature of the continuous film. Corresponding Raman spectra is shown in Figure 2e for eight randomly picked points along the direction of the line scan. The full-width at half-maximum (FWHM) as calculated from these traces are ~4.5 and ~6 cm^−1^ for the E^1^_2g_ and A_1g_ peaks, respectively, indicating good crystalline quality of the grown MoS_2_.

A second Raman line scan was performed to determine the composition of the thick white material grown in zone A. Figure 2f,h shows the line mapping results obtained over a 0.5 mm distance, marked by the black arrow in Figure 2a. In contrast to the results of Figure 2g, the frequency difference between the E^1^_2g_ and A_1g_ peaks measured in this region is found to have a significantly higher value of ~26 cm^−1^, and therefore corresponds to bulk MoS_2_. As we additionally prove in the following discussions, using spectra collected over a wider wavenumber range, the thick material found in this region represents fully sulfurized bulk MoS_2_ and does not contain any intermediate by-products such as MoOS_2_. It must be noted that, in contrast to the overlapping Raman traces seen in Figure 2e, in Figure 2f there instead appears to be a random spread in the intensities across the mapped line (see color contour shown in Figure 2h). We believe these intensity variations are directly related to the thickness variations of the bulk MoS_2_ across the scanned locations. Similar intensity variations have been observed previously and were attributed to optical interference effects arising from the presence of the underlying oxide layer [55].

The formation of thick MoS_2_ material grown in zone A can be attributed to the high concentration of Mo-flux arriving at the leading edge of the substrate. As nucleation progresses further away from the sample edge, the nucleation density lowers and becomes favorable for the growth of triangular domains and their subsequent coalescence [23]. This ultimately results in zone C being covered with a continuous MoS_2_ film.

### 3.2. Effect of MoO_2_ Precursor Quantity

**Figure 3:**
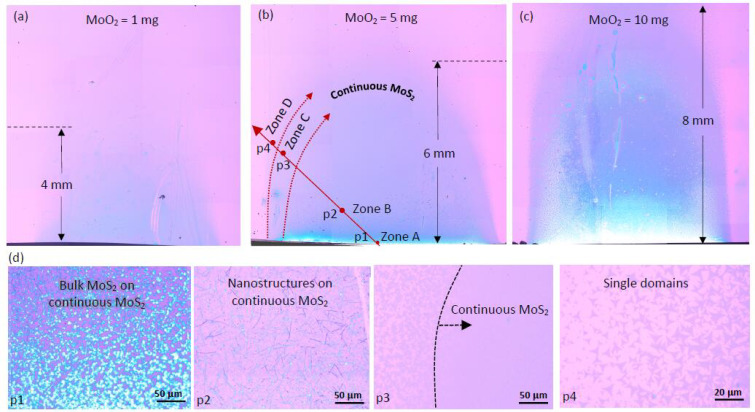
Effect of MoO_2_ precursor quantity on MoS_2_ growth. High-resolution optical stitched image capturing the MoS_2_ growth results obtained when (**a**) 1 mg, (**b**) 5 mg and (**c**) 10 mg of MoO_2_ precursor is used. Growth parameters fixed for this experiment were S = 300 mg, gas flow rate = 100 sccm, growth temperature = 760 °C and growth time = 1 min. (**d**) Zoomed in optical images showing the different growth morphologies taken at corresponding points p1–p4 marked in (**b**).

To demonstrate the significance of the Mo-flux, we performed an experiment in which the MoO_2_ quantity was varied while the gas flow rate was held constant at a high value of 100 sccm (Figure 3). The diffusion of MoO_2_ vapor from the source to substrate can be quantified by using a common solution to the diffusion equation [56]:

(8) $$n\left(x,t\right)\;=\;n\left(0,t\right)\;exp\left[-\frac{x^{2}}{4Dt}\right]$$

where *t* is the time, *D* is the diffusion constant and n(0,t) and n(x,t) are the concentrations of the gaseous species at the source and a distance *x* away from the source, respectively. Evidence of the n(0,t) dependency can be seen experimentally from the growth results shown in Figure 3a–c. Here, a systematic expansion of the growth zones is observed as the amount of MoO_2_ precursor is increased from 1 mg to 5 mg and 10 mg. For the case when 10 mg of MoO_2_ is used, the outermost growth zone extends all the way across the substrate length. Although this increase in wafer coverage may be viewed as beneficial, the simultaneous strengthening of the concentration gradient is undesirable as it results in more regions of the wafer being covered with thick MoS_2_ product, as clearly visible from the bright contrasted regions towards the upstream end of the substrate (Figure 3c). On the contrary, while the gradient produced by the smallest MoO_2_ quantity (1 mg) is not immediately apparent, the wafer coverage is evidently poor.

Figure 3d shows zoomed-in images captured at the locations p1 through p4, marked in Figure 3b. Each image captures the growth morphology in the respective zones A to D (also marked in Figure 3b). An accidental scratch mark at location p2 revealed that the nanostructures discovered at this location were actually formed on top of a continuous bottom layer. This was later found to be true for the bulk MoS_2_ crystals grown in zone A, as well.

### 3.3. Effect of Gas-Flow Rate on MoS_2_ Morphology

**Figure 4:**
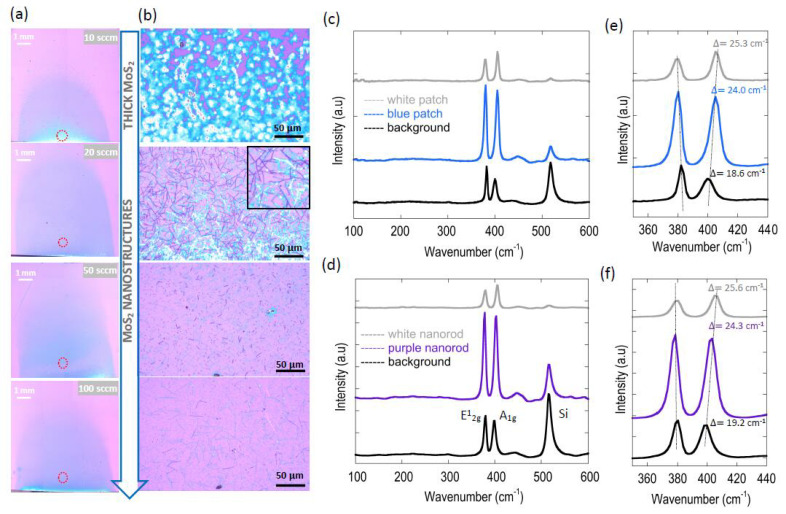
Effect on flow rate on MoS_2_ grown in Zone A. (**a**) High resolution optical stitched image showing MoS_2_ growth results at several Ar gas flow rate settings of (**a**) 10 sccm, (**b**) 20 sccm, (**c**) 50 sccm and (**d**) 100 sccm. The remaining growth parameters for this study were MoO_2_ = 5 mg, S = 300 mg, growth temperature = 760 °C and growth time = 1 min (**b**) Zoomed in optical images capturing MoS_2_ growth morphologies at corresponding locations in (**a**) marked by dotted red circles. (**c**) Raman spectra acquired over a wide spectral range to reveal compositions of the blue- and white-colored structures, as well as the background film material grown in zone A at the gas flow rate of 10 sccm. (**d**) Similar spectra as (**c**), but for the purple- and white-colored nanorods, as well as the background film material grown in zone A at the gas flow rate of 20 sccm. (**e**,**f**) Zoomed-in versions of spectra shown in (**c**,**d**), respectively, capturing the positions of the E^1^_2g_ and A_1g_ vibration modes. The dotted lines are a guide to the eye, showing the increasing peak difference values as detected material gets thicker.

**Figure 5:**
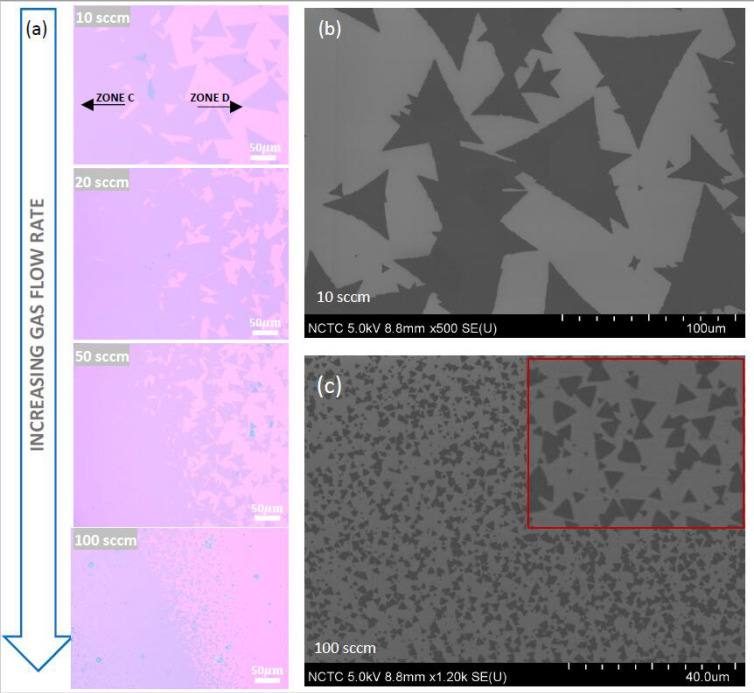
Effect of flow rate on the average size of the MoS_2_ domains grown in the outermost growth zone D. (**a**) Optical images showing systematic increase of average domain size at the boundary separating zones C (continuous film) and D (individual domains). Growth parameters fixed for this experiment were S = 300 mg, MoO_2_ = 3 mg, growth temperature = 760 °C and growth time = 1 min. (**b**,**c**) FESEM micrographs capturing average domain size for the 10 sccm and 100 sccm flow rate experiments, respectively. The inset to (**c**) is a 5× zoomed image of a small area in the micrograph shown in the main panel.

**Figure 6:**
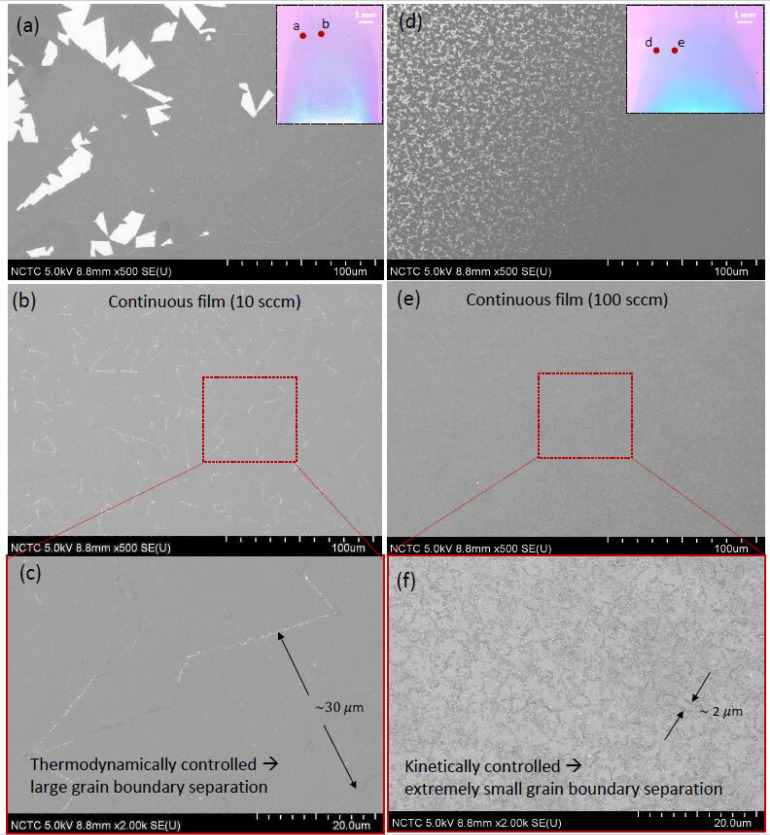
FESEM micrographs capturing the drastic morphology variation in the grown continuous monolayer MoS_2_ films as the gas-flow-rate condition is changed from (left panels) 10 sccm to (right panels) 100 sccm. (**a**,**d**) Micrographs obtained at the boundary separating zones C and D for the gas-flow-rate condition of 10 and 100 sccm, respectively. The insets show the optical image of the growth pattern at the approximate locations (marked in red) at which the SEM scan was performed. (**b**,**e**) Micrographs similar to (**a**,**d**) taken at a location well inside zone C. (**c**,**f**) High-magnification micrographs of (**b**,**e**), revealing details of the grain boundary separations in the grown continuous MoS_2_ layers.

To further explore the aforementioned phenomenon, we performed a detailed investigation of the effect of gas-flow rate on growth conditions, the results of which are presented in Figure 4, Figure 5 and Figure 6. The immediate observation from the stitched optical images shown in Figure 4a is that close to the leading edge of the growth substrate (marked in Figure 4a) the concentration gradient is seen to be significantly dampened as the flow rate is increased. Interestingly, the growth of nanostructures was observed for growth using higher gas flow rates, as evident from the optical images corresponding to the gas flow rate conditions of 20, 50 and 100 sccm (see Figure 4b). For the lowest flow-rate condition of 10 sccm, these nanostructures are largely absent, and the growth was dominated by thick MoS_2_ material.

Similar nanostructures have been reported in prior works [29,53,57,58], using an MoO_3_ precursor. According to Vila et al. [53], depending on the MoO_x_:S_2_ partial pressure available at the growth site, these nanostructures can be fully MoO_2_, partially sulfurized MoO_2_ or even MoS_2_. Therefore, to verify the structural composition of the thick material and the nanostructures, we performed Raman analyses for each of these cases. As can be seen from the data shown in Figure 4c,d, the Raman scan obtained in a wide wavenumber range of 100–600 cm^−1^ revealed only two peaks (A_1g_ and E^1^_2g_), which are characteristic of MoS_2_ material, in addition to the expected peak of Si seen at a wavenumber of 516 cm^−1^. There were no peaks corresponding to MoO_2_ or MoOS_2_ at any of the six locations analyzed, indicating that complete sulfurization of MoO_2_ occurred. Interestingly, the Raman traces obtained from the background revealed that these regions were covered with a continuous film of monolayer MoS_2_ (peak differences of 18.6 and 19.2 cm^−1^ for 10 and 20 sccm cases, respectively), indicating that the thicker material and nanostructures could have grown after the formation of an initial layer of monolayer MoS_2_. Such a “layer-plus-island” growth mode may indicate that the surface and edge energy (ΔE) changes from initially being negative (ΔE < 0) to taking positive values (ΔE ≥ 0) later [53,59].

Figure 4e,f show the corresponding spectrum of Figure 4c,d, respectively, but for a narrower spectral range. Here the thickness difference between the detected locations is evident. For example, while the background film exhibited a peak difference of 18.6 cm^−1^, confirming its monolayer nature, the blue- and white-colored patches exhibited peak difference values of 24 and 25.3 cm^−1^ respectively, and therefore corresponds to significantly thicker/bulk MoS_2_ material. Similar observations are also made for the nanostructures that were grown at the gas flow rate condition of 20 sccm (see Figure 4f).

Surprisingly, as seen in the various panels of Figure 4a, the bright patches at the leading edge of the substrate do not systematically dampen as the flow rate increases. For example, while we observe no regions of bulk MoS_2_ formation at the flow rate of 20 sccm, these regions seem to reappear at higher flow rates of 50 and 100 sccm. This rather unexpected result can be explained in terms of the substrate edge driving changes in the nucleation process [23]. Because the substrates were individually cleaved prior to growth, it is conceivable that the morphology of the edges could differ dramatically from one substrate to the next, resulting in this undesirable yet unavoidable effect on the ultimate growth result.

### 3.4. Effect of Gas-Flow Rate on Domain Size and Grain-Boundary Separation of Continuous MoS_2_

In addition to creating favorable growth conditions for the formation of nanostructures, the flowrate also plays a significant role in determining the average size of the individual MoS_2_ domains grown in zone D. As discussed in the following sections, this is a very important parameter that determines the quality of continuous films found in zone C. As the flow rate is increased from 10 to 100 sccm, the average domain size is reduced dramatically, from ~70 μm to less than 2 μm (see Figure 5a). The FESEM scans comparing the 10 and 100 sccm case are shown in Figure 5b,c, respectively. These results are in agreement with previous studies on the growth of graphene domains [60], where it was noted that a lower flow rate suppresses the nuclei density, which then facilitates the growth of larger domains. Z Lin et al. [26] reported a similar effect on nuclei density dependent domain sizes for MoS_2_ grown by using MoO_3_ precursor; however, the change in nuclei density reported in their work was due to the change in relative position of the substrate to the precursor source.

Given the dramatic effect on the domain size on the gas flow rate, it is imperative to investigate the surface morphologies of the continuous films. Figure 6 shows a comparison of FESEM images of the MoS_2_ layers grown at a flow rate of 10 sccm (Figure 6a–c) and 100 sccm (Figure 6d–f). Figure 6a,d captures the images at the boundary separating the continuous films and the individual domains (see insets identifying the location scanned). The magnitude of the difference in domain sizes between low and high flow rates is striking, as can be seen here. Figure 6b,e captures similar magnification images of those shown in Figure 6a,d, but for an area containing only the continuous film. For the 10 sccm flow rate condition, the grain boundaries are immediately visible and a zoomed in image (Figure 6c) reveals that, on an average, the grain boundary separations range between 20 and 30 μm, with certain regions even exceeding these values. The thickness uniformity over several tens of micrometers is made possible by the growth of large domain sizes, which indicates the high quality of the grown films [23,61]. On the other hand, the high-flow-rate condition of 100 sccm severely affects sample quality. While a lower magnification SEM image (panel (e) of Figure 6) reveals what may appear to be a continuous film without any grain boundaries, careful inspection at a higher magnification (panel (f) of Figure 6) proves, without doubt, the existence of extremely small grain (~2 μm) boundary separations in the grown film.

These results signify that an increased flow rate speeds up the mass transfer process, resulting in a faster crystal growth rate [24]. The latter introduces a high level of instability in the growth process by disallowing atoms to move into (and grow freely) at locations with the lowest surface free energy, leading to a so called “kinetically controlled” growth condition in contrast to a thermodynamically controlled one that is favored under low flow rate conditions.

In addition to the flow rate’s effect on the concentration gradient and the domain sizes, it is also noted from Figure 4a that substrate coverage can be improved with a higher flow rate (compare 10 and 20 sccm); however, a limit is apparently reached as we approach the highest flow rate of 100 sccm explored here. A tradeoff therefore exists between achieving better wafer coverage versus obtaining continuous films containing larger domain sizes. A strategy to improve wafer coverage is described in the later sections.

### 3.5. Influence of Critical Growth Parameters on MoS_2_ Domain Shape and Size

**Figure 7:**
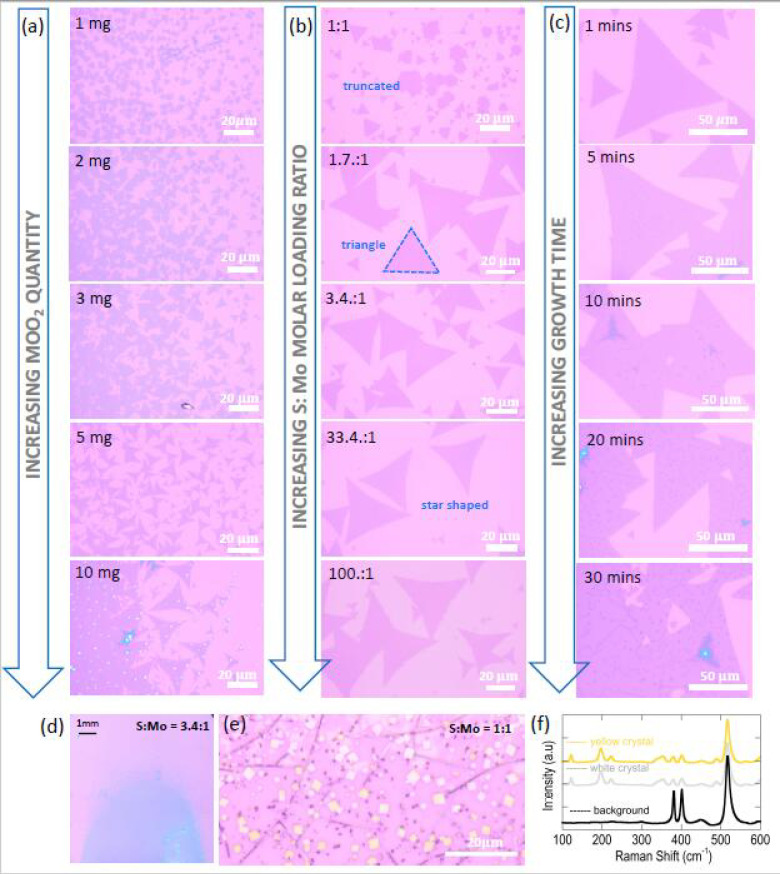
Influence of growth parameters on domain size, shape and morphology. (**a**) Optical images capturing the increase of MoS_2_ domain size as the MoO_2_ precursor quantity is increased from 1mg to 10 mg. Growth parameters fixed for this experiment were S = 300 mg, gas flow rate = 100 sccm, growth temperature = 760 °C and growth time = 1 min. (**b**) Optical images showing the shape of the formed MoS_2_ domains varying from truncated to triangular to star-shaped as the S:Mo molar loading ratio is increased from 1:1 to 100:1. Growth parameters fixed for this experiment were MoO_2_ = 3 mg, gas flow rate = 10 sccm, growth temperature = 760 °C and growth time = 1 min. (**c**) Optical images showing the formation of nanoparticles on the surface of the formed MoS_2_ domains as the growth time is increased from 1 min to 30 min. Growth parameters fixed for this experiment were MoO_2_ = 5 mg, S = 300 mg, gas flow rate = 10 sccm and growth temperature = 760 °C. (**d**) High-resolution optical stitched image capturing the MoS_2_ growth results at a molar loading ratio of 3.4:1 (**e**) Optical image showing the formation of intermediate state at the lowest S:Mo loading ratio of 1:1. (**f**) Raman traces obtained for the white- and yellow-colored crystal and also the background film.

It should be clear by now that a thorough understanding of how domain attributes might be adjusted to achieve desirable qualities in the continuous films is required. We have therefore performed additional tests to explore the impacts of MoO_2_ amount, S amount and growth duration (Figure 7).

#### 3.5.1. Domain-Size Dependency on MoO_2_ Precursor Amount

In addition to tuning domain size by using flow rate, the quantity of MoO_2_ precursor used also plays an important role. This can be seen in Figure 7a, which reveals a systematic increase in domain size as the precursor amount is increased. For a MoO_2_ quantity of 10 mg, the largest domain sizes are obtained. However, it must be recalled from the results of Figure 3c that a MoO_2_ quantity increase is also associated with a much stronger concentration gradient across the length of the sample. Unfortunately, this means that the domains formed are not homogenous and could potentially contain regions of thick MoS_2_, as is rightly revealed by the optical image taken for the 10 mg case (Figure 7a). In this regard, there is a trade-off in that the amount of MoO_2_ precursor used must be carefully chosen in order to achieve the required gradient distribution and domain sizes.

#### 3.5.2. Domain Shape Dependency on S:Mo Molar Loading Ratio and Identifying the Regime of Intermediate-State-Free Growth of MoS_2_

Turning our attention to domain shape, we can observe from the growth results already shown in Figure 5a that the formed MoS_2_ domains are closer to a star shape rather than triangular. This is a well-known consequence of the presence of a S-rich growth condition [24,31], which, in our case, was created by the high value of S:Mo molar loading ratio of 100:1 set for the experiment. However, if desired, the domain shape can be tuned by lowering the S:Mo ratio until the stoichiometric requirement is reached to form perfect triangular domains. This is evident from Figure 7b, where a systematic evolution from a star shape to triangular domain is observed as molar loading ratio S:Mo is lowered in steps from 100:1 to 1.7:1. When the loading ratio is further reduced to 1:1, there are two significant deviations in the growth result. First, we see much smaller triangular domains along with truncated triangles in the mix. The formation of truncated domains is consistent with what is expected for a S-deficient condition and has been reported previously by other groups [23,24,31,34] that have used traditional MoO_3_ as the precursor. Second, and perhaps most intriguingly, we discovered how this S-deficient condition resulted in the formation of intermediates near the substrate’s leading edge (Figure 7e). While this effect was most pronounced for a loading ratio of 1:1, trace amounts with much smaller crystal sizes were also seen at a loading ratio of 1.7:1. Raman spectra shown in Figure 7f confirmed these crystals to be MoOS_2_. The point analysis that was performed on the background film revealed that it was monolayer MoS_2_ with a peak difference of ~20 cm^−1^. These findings imply that there may be competing mechanisms of growth involving formations of both MoS_2_ and MoOS_2_, especially when the growth conditions become S-deficient.

It is worth noting that, with the exception of the lowest S:Mo molar loading ratios of 1:1 and 1.7:1 (intermediates almost unnoticeable at this value), no intermediate state formations were observed from careful optical inspection of the MoS_2_ material grown from all other S:Mo conditions. As matter of fact, both MoO_2_ and MoOS_2_ are known to have very distinct morphologies (rectangular domains) when compared to MoS_2_, and they can therefore be easily identified on a substrate, using simple optical microscopy. The rather interesting revelation from our findings is in contrast to findings of previous experiments employing MoO_3_ as the precursor. To provide further validation, a few examples can be taken from the literature. For instance, Pondick et al. [36] reported that trace amounts of intermediate MoOS_2_ states were formed at the leading edge of the substrate, even with a globally set molar loading ratio as large as 187:1. Najmaei et al. [23] reported that a sulfur concentration greater than 500 mg was needed to avoid oxysulfide formations. Similarly, in Reference [25], a molar ratio greater than 170:3 was required to avoid intermediate products.

For MoOS_2_ to fully form from the reaction dictated by Equation (3) [36], the S:Mo ratio available for reaction must be 2.5:1. Likewise, to completely form MoS_2_ from the direct VPS of MoO_2_, the stoichiometric requirement is 3:1 (see Equation (7)). Therefore, to be sure that the intermediate reactions can be bypassed, a minimum S:Mo loading ratio of 3:1 should be used for reliable growth of intermediate-free MoS_2_, which is consistent with our experimental findings (an example of the growth pattern obtained at a molar ratio of 3.4:1 is shown in Figure 7d). Furthermore, because the outcome of the growth can be explained directly in terms of the global loading ratio set by the experiment, this could indicate a much closer agreement between the globally set S:Mo ratio and the locally available S:Mo ratio for reaction at the substrate. In this context, the single step VPS of MoO_2_ may provide a more effective means to control CVD growth parameters when compared with the traditional route of growing MoS_2_, using the VPS of MoO_3_.

Having highlighted these significant differences, it should be noted that a direct comparison of the molar ratio dependence reported in this work with those obtained by others should be approached with caution, because experimental conditions of the CVD setup can vary greatly. Importantly, because the local S:Mo ratio is the ultimate factor deciding the outcome of the growth material, geometrical factors must also be considered. For example, while the distance between the S and the growth substrate set in Reference [36] is close to the value used in our study, the CVD tube size was significantly different (1-inch o.d. in Reference [36] compared to 2-inch o.d. in our study).

#### 3.5.3. Influence of CVD Growth Time on Nanoparticle Formations

In Figure 7c we show the effect on the domains when increasing the growth time from 1 min to a maximum of 30 min. The key consequence observed here is an acceleration of the creation of self-seeding nucleation sites (nanoparticles) on top of the MoS_2_ domains, as growth time increases. The nanoparticles start to appear as early as 5 min and systematically increase in density as the growth time is increased. Similar nucleation sites formed on top of MoS_2_ domains have been reported previously [37,62]. In Reference [37], the self-seeding mechanism was reported to be caused by increasing growth temperature, while in Reference [62], the increase of growth time has been shown to cause the same effect. We found that when the growth time is limited to 1 min, these nanoparticles are typically not present. This allowed us to grow materials that are more homogenous (see Figure 2a and Figure 5a) and have a reduced risk of additional layer formations stemming from these seeds. In prior work by Bilgin et al. [17] the rapid formation of second layers was reported, albeit at a much higher growth temperature of 950 °C. In addition to the inherent acceleration provided by the absence of intermediate state formations in the direct VPS of MoO_2_, this finding was attributed to the faster growth kinetics resulting from the higher temperature used for the growth. While the MoS_2_ domains reported in our work commonly revealed domain edge irregularities (expected for S-rich conditions), we do not observe any significant secondary layer formation when the domains are viewed under optical contrast, SEM imaging or AFM measurements. We attribute this to both the lower temperature (760 °C) and the short growth time employed in our work. This is in agreement with earlier findings [17] and emphasizes the important role of the growth temperature. While we did not conduct a comprehensive study of the effect of growth temperature in this work, we found that lowering the temperature below 700 °C resulted in a low MoS_2_ yield with no continuous monolayer formation. For growth temperatures greater than 800 °C, however, we found that the grown material was thick and non-homogeneous throughout the growth zones. Monolayer MoS_2_ (typically found in zones C and D) was consistently obtained when the growth temperature was set to an intermediate temperature of ~760 °C, which led us to select this temperature as the optimal growth temperature in the current study. While these findings indicate a strong temperature dependence of nucleation density, more detailed experiments (not performed here) examining changes in film growth with systematically varying the growth temperature will be required to fully comprehend the intricate growth mechanisms involved.

### 3.6. Effect of Precursor Spatial Distribution on Wafer Coverage

While we believe that the results presented in this work are a significant step forward, we have not yet demonstrated wafer-scale growth and/or wafer-scale uniformity of continuous monolayer MoS_2_. In a recent work employing MoO_3_ precursors [49], two kinds of growth regimes were identified: a diffusion regime and a surface-reaction regime, which corresponded to whether a large or small amount of MoO_3_ precursor was used for the growth, respectively. While in the diffusion regime, a gradient-like growth pattern, similar to those found in this work, was observed, in the surface reaction regime, the growth of continuous films with more than 90% wafer coverage was reported. The authors have attributed this to the reduction of the gas-phase reaction facilitated by the low MoO_3_ precursor quantity used and the low-pressure CVD growth conditions. In our experiments, we do not seem to transition into the surface-reaction mode, despite performing experiments with a very low (1 mg) MoO_2_ quantity (see Figure 3a). The reasons for this are not fully clear; however, in addition to the obviously different CVD process used (APCVD vs LPCVD), other geometrical factors, such as the source to substrate gap height/distance, could also play a significant role in limiting growth to the diffusion regime.

While further studies are warranted, a few other strategies to improve wafer coverage can also be considered. For instance, wafer-scale growth was very recently reported by Q Wang et al. [47] Here a specially designed multisource tube furnace was employed which allowed for a uniform distribution of Mo precursors to arrive at the growth substrate for nucleation. Other approaches, including the strategic introduction of S [45], increasing the substrate-source gap height [26], orienting the substrates vertically [28], and using barriers [48] have resulted in continuous films. In a much earlier work by Najmei et al. [23], the strategic placement of step edges across the growth substrate was also shown to promote uniform nucleation over a large area.

**Figure 8:**
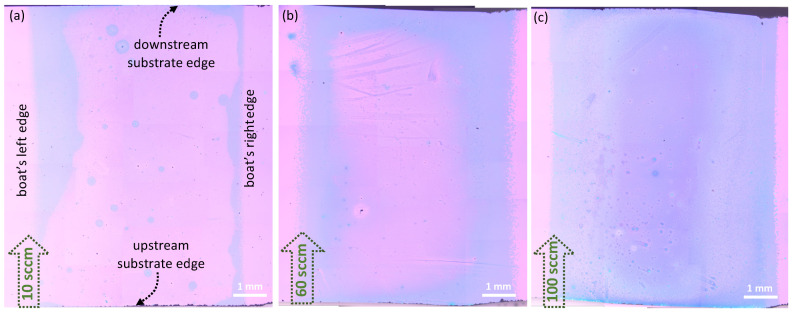
Strategy to improve wafer coverage by spreading MoO_2_ precursor uniformly in the boat. High-resolution optical stitched images showing improvement in the percentage of wafer coverage for different gas flow rate conditions of (**a**) 10 sccm, (**b**) 60 sccm and (**c**) 100 sccm.

Here, we present a different approach that provided a modest improvement in wafer coverage. In an attempt to create a uniform distribution of Mo species with minimal to no gradient, we spread the MoO_2_ precursor uniformly across the entire length of the boat. Figure 8a shows the growth results obtained for such a scenario, while keeping all other growth parameters unchanged. A distinct pattern of film growth can be seen here, completely different from the zonal patterns observed for all other cases when the precursor was simply placed as a heap at a determined location on the boat. Interestingly, from Figure 8a it is evident that the growth of these films tends to initiate from all four edges of the substrate and then progress towards the center of the wafer, although not quite reaching there. This observation once again proves the strong influence that substrate edges and defects could possibly have in determining the final outcome of the growth processes, strongly in agreement with earlier findings by Najmei et al. [23]. We also found that, by increasing the flow rate, we could achieve improved wafer coverage (see Figure 8b,c), albeit with poor film thickness and morphology uniformity. For instance, a flow rate of 100 sccm yielded an entire substrate covered with growth material. However, as already noted, the higher flow rate drives the reaction towards unstable conditions and results in the formations of undesirable nanostructures on top of the MoS_2_ film. Additionally, we found it very difficult to achieve a uniform spread of the MoO_2_ across the boat, mainly due the small amounts of MoO_2_ used, and the fact that this step was being performed manually. Due to these reasons, no solid conclusions can be made at this point about the efficacy of this strategy, but we believe the insights derived from this paper may be beneficial for future works.

## 4. Conclusions

In conclusion, we have successfully demonstrated the possibility of growing continuous films of monolayer MoS_2_, using the single-step VPS of MoO_2_. The growth results exhibited a predominant zonal pattern which is typical of the presence of a varying concentration-gradient of the Mo-source impinging on the substrate surface. By employing a high-resolution optical-image-stitching approach, we could identify four distinct growth zones. At the upstream end of the substrate, growth was dominated by thick materials and “rod-like” nanostructures grown on top of continuous MoS_2_, while a cm-scale continuous film of monolayer MoS_2_ was grown in the downstream location. This was followed by the growth of individual MoS_2_ domains at the outermost growth zone.

The continuous MoS_2_ films grown in this work had a large area and were homogenous, as confirmed by Raman line map analysis. More importantly, the grain size of domains coalescing into continuous layers was found to be a strong function of the gas-flow rate used in the experiment. An order of magnitude reduction from ~30 to ~2 μm in grain-boundary separations was observed from SEM micrographs, as the gas flow rate was increased from 10 to 100 sccm, respectively, which meant that higher-quality MoS_2_ films were grown at the lowest gas-flow-rate condition. The dramatic effect of the gas-flow rate suggests that, as the gas-flow rate is increased, the growth process transitions from a thermodynamically controlled and stable process to a kinetically controlled and unstable one.

A significant finding of this work is that, for a wide range of S:Mo loading ratios investigated, the material synthesized on all identified growth zones was found to be solely MoS_2_, as confirmed by extensive Raman analyses, with no additional Raman peaks corresponding to any intermediate states, such as MoOS_2_ or MoO_2_. This is in striking contrast to previous reports using MoO_3_ as the precursor, where the formation of intermediate MoOS_2_ states was found to be a significant outcome of the growth process, even at excessively high S:Mo molar loading ratios. This further confirms earlier assertions of the efficacy of using a single-step VPS method for synthesizing MoS_2_. Our experiments reveal that the formation of intermediate states are highly disfavored, as long as the global S:Mo molar ratios are kept above the value of 3:1, which is stoichiometrically required by the VPS reaction of MoO_2_.

Finally, while we have yet to demonstrate wafer-scale continuous MoS_2_ growth by using the direct VPS of MoO_2_, a strategy to improve coverage via uniform distribution of the MoO_2_ source was tested and appeared to yield promising results. However, more work is needed to improve the homogeneity of the grown films. We believe that the simple reaction mechanics of the VPS of MoO_2_ and the associated high level of safety of using this material as the precursor make this a promising avenue for realizing wafer-scale monolayer MoS_2_ that is not only of high quality but also compatible with existing CMOS standards.

## Data Availability

The data presented in this study are openly available in FigShare at https://doi.org/10.6084/m9.figshare.15168456.
